# Unmasking Invisible Burdens: A Systematic Review on Perceived Discrimination and its Impact on Allostatic Load in Black Individuals

**DOI:** 10.1002/alz.086938

**Published:** 2025-01-09

**Authors:** Alaa Harb, Ana W. Capuano, Lisa L. Barnes, Juliana Nery Souza‐Talarico

**Affiliations:** ^1^ University of Iowa, Iowa City, IA USA; ^2^ Rush University Medical Center, Chicago, IL USA

## Abstract

**Background:**

Prolonged discrimination is psychosocial stressor, influencing mortality rates and contributing to cardiovascular and mental health disorders among Black individuals. Allostatic load (AL), the wear and tear of stress is a biological cumulative risk that links psychosocial stressors to adverse health outcomes. Currently, a consolidate review of evidence underscoring discrimination and AL in Black individuals is not available. The purpose of this review is to synthesize the existing evidence on the association between perceived discrimination and allostatic load (AL) in Black individuals, and to examine whether this relationship may underlie racial health disparities.

**Method:**

We searched six databases (PubMed, CINAHL, EMBASE, PsycINFO, Web of Science, and Scopus) and completed manual searches of published studies to obtain potentially relevant data. Two independent reviewers assessed studies for original research measuring discrimination (i.e., validated scales) and AL (i.e., biomarkers of stress‐target systems) in Black individuals 18 years and older.

**Result:**

This review included 11 studies with a total of 24,087 participants. The majority were Black females between 18‐96 years old, although 3 studies had exclusively Black samples. Perceived discrimination was measured using everyday and lifetime discrimination scales. Allostatic load was assessed via blood biomarkers representing cardiovascular, metabolic, immune, and neuroendocrine systems. Across studies, higher reported discrimination was associated with elevated allostatic load, indicating greater physiological dysregulation. This relationship persisted after adjusting for potential demographic and health confounders. However, findings were mixed regarding the specific biomarkers related to discrimination, and a few studies found no significant association.

**Conclusion:**

The available evidence suggests a correlation between perceived discrimination and heightened allostatic load in Black individuals, which may help explain racial health disparities. However, inconsistencies across studies indicate a need for additional longitudinal research to clarify the relationships between discrimination, physiological stress responses, and health outcomes over time. Elucidating these mediating processes can inform interventions aimed at mitigating the adverse effects of discrimination on minority health.

